# Case report: Myocardial tuberculosis-MRI

**DOI:** 10.4103/0971-3026.45347

**Published:** 2009-02

**Authors:** Rashmi Dixit, Veena Chowdhury, Sapna Singh

**Affiliations:** Department of Radiodiagnosis, Maulana Azad Medical College, New Delhi, India

**Keywords:** Heart, MRI, myocardium, tuberculosis

Although tuberculosis can affect any organ in the body, the respiratory tract is the organ most commonly affected. Myocardial tuberculosis is extremely rare.[1] We report the MRI features of myocardial tuberculosis in a patient initially diagnosed to have a right atrial mass.

## Case Report

A 30-year-old woman was admitted with palpitations, easy fatigability, and breathlessness of 2 months' duration. On examination, the patient was febrile, the heart rate was 100/min, the blood pressure was 120/80 mm Hg, and the jugular venous pressure was raised. Small matted lymph nodes were palpable in the neck. There was hepatomegaly, but no peripheral edema or ascites. Blood investigations revealed a hemoglobin level of 10 gm% and normal white blood cell counts. The Mantoux test was positive (20 mm). The chest radiograph showed cardiomegaly, with no specific chamber enlargement; there were patchy opacities in the right mid-zone.

**Figure 1:**
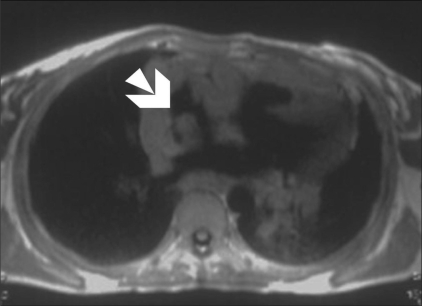
T1W axial MRI image shows diffuse myopericardial thickening, with the thickened myocardium projecting into the right atrium (arrow)

**Figure 2:**
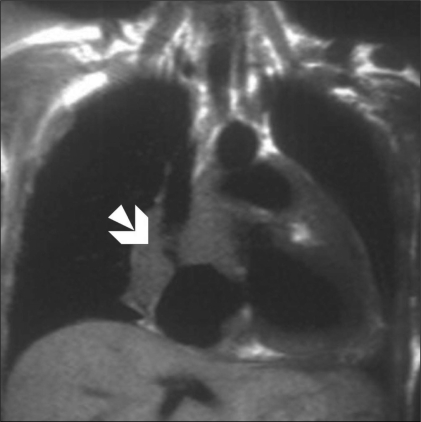
T2W dark-blood coronal MRI image shows diffuse myopericardial thickening. The thickening is hypointense on T2W images also and is causing attenuation of the proximal SVC (arrow)

**Figure 3:**
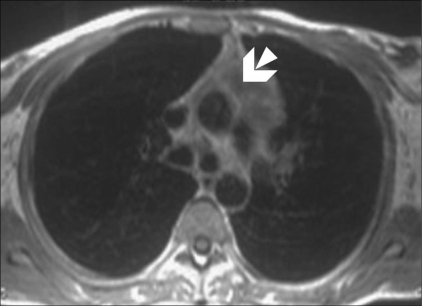
Postcontrast axial T1W MRI image shows peripherally enhancing lymph nodes (arrow) in the prevascular space

**Figure 4:**
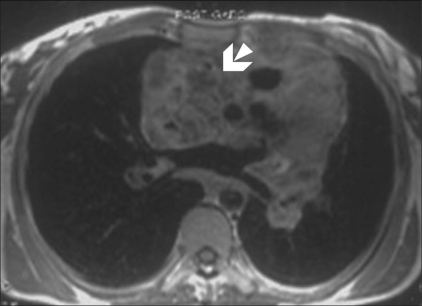
Postcontrast axial T1W MRI image also shows conglomerate nodular and ring enhancement (arrow) of the markedly thickened RA myocardium

Echocardiography revealed normal left ventricular function, intact interatrial and interventricular septa, no pericardial effusion, and a hypoechoic nodular mass in the right atrium, which was reported to be either a neoplasm or a thrombus. Cardiac MRI was performed with ECG-gated T1W, T2W, and postcontrast sequences obtained in multiple planes. Standard cardiac MRI sequences using balanced gradients, e.g., TrueFISP, were performed. Cine images were also acquired. Cardiac MRI showed thickened myocardium with indistinct planes between the myocardium and the pericardium. Marked nodular thickening was noted at the right atrium–superior vena cava (SVC) junction, which was projecting into the right atrial cavity [Figure 1]. Attenuation of the proximal SVC and dilatation of the inferior vena cava (IVC) were also noticed. The lesion appeared iso- to hypointense on both T1W and T2W images [Figure 2]. Multiple, enlarged mediastinal lymph nodes were also noted [Figure 3]. The contrast-enhanced images revealed heterogeneous enhancement of the entire myocardium [Figure 4] with conglomerate ring-like and nodular enhancement in the region of the right atrium–SVC junction, with compression of the SVC. The myopericardium-pericardium interface was indistinct. The mediastinal nodes also showed peripheral enhancement with nonenhancing centers.

These imaging features were nonspecific, and both infective and neoplastic conditions were considered in the differential diagnoses. In view of the T2 hypointensity of the lesion, as well as the peripheral enhancement within the lesion and the enlarged mediastinal nodes, the possibility of tuberculosis was also considered.

**Figure 5:**
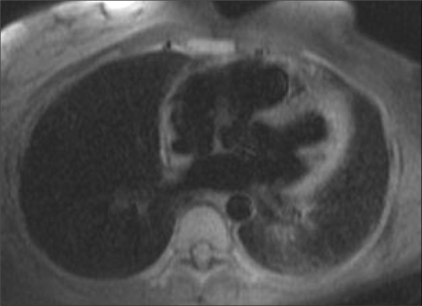
Posttreatment T1W axial MRI image reveals normal appearance of the myopericardium and regression of the mediastinal lymphadenopathy

**Figure 6:**
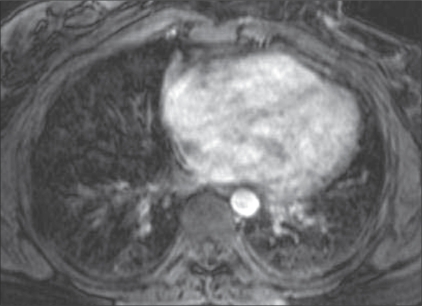
Posttreatment, postcontrast axial T1W MRI image after treatment reveal no abnormal enhancement of the myopericardium

Fine needle aspiration cytology from one of the cervical lymph nodes revealed caseating granulomas. Although the evidence was circumstantial, the patient was assumed to have myocardial, pulmonary, and nodal tuberculosis and was given antituberculous therapy. A follow-up MRI done 12 months later revealed complete resolution of the myocardial abnormality and the mediastinal lymphadenopathy [Figures 5 and 6]. The patient became asymptomatic after 12 months and there was complete resolution of the lung lesions.

## Discussion

Myocardial tuberculosis is considered an uncommon clinical entity and is only infrequently diagnosed antemortem.[2] Postmortem studies show that isolated myocardial involvement is responsible for < 0.1% of tuberculosis-related deaths.[3] Most cases occur secondary to hematogeneous spread from a remote tuberculous focus, lymphatic spread from mediastinal nodes, or direct spread from the adjacent pericardium.[2,4]

Pathologically, there are three patterns of involvement;[4] these are as follows: (i) miliary tuberculosis, with the heart being just one of the many organs involved; (ii) diffuse infiltrating interstitial disease; and (iii) caseating nodular disease (tuberculoma). The miliary form is by far the commonest; tuberculomas are much rarer.[4] The lesions may involve the atria, the ventricles, or the interventricular septum.[5] Tuberculosis may lead to heart block[6] or other abnormal rhythms, as well as myocardial rupture.[7] The diagnosis rests on the typical histological changes.[2]

Typically, echocardiography may show an echogenic immobile mass in the ventricular myocardium in the case of a calcified myocardial tuberculoma.[8] Cardiac MRI is a recent tool and shows a characteristic T2 shortening in tuberculous involvement, similar to that seen in intracranial tuberculomas.[9] The characteristic appearance on T2W images includes a central isointense core, corresponding to central caseation; a hypointense rim, which represents the fibrous capsule; and a thin hyperintense line, which correlates with an inflammatory cellular infiltrate.[9] Post-gadolinium MRI in our patient showed ring enhancement with conglomeration. Conglomeration is due to the presence of a granulomatous lesion and is usually not seen in neoplasms.[9]

Both echocardiography and MRI findings are often nonspecific in cardiac masses. The differential diagnosis in such cases includes right atrial myxoma and thrombus.

In our case, the T2 hypointense character of the diffuse myocardial involvement, including the nodular lesion at the right atrial inlet, and the extracardiac findings of necrotic mediastinal nodes, led us to suspect the possibility of tuberculosis. Regression of disease has been reported in response to antituberculous therapy[8,10,11] and this was also seen in our case. In cases where the diagnosis is still doubtful, surgical resection with biopsy may be required.[12]
